# Sudachinoid- and Ichangensin-Type Limonoids from *Citrus junos* Downregulate Pro-Inflammatory Cytokines

**DOI:** 10.3390/ijms21186963

**Published:** 2020-09-22

**Authors:** Jihun Shin, Hwa Young Song, Mina Lee

**Affiliations:** College of Pharmacy and Research Institute of Life and Pharmaceutical Sciences, Sunchon National University, Suncheon 57922, Korea; wlgns0551@s.scnu.ac.kr (J.S.); blueocean33@s.scnu.ac.kr (H.Y.S.)

**Keywords:** *Citrus junos*, limonoids, inflammation, interleukin-1β, nuclear transcription factor κB, inflammatory bowel disease

## Abstract

Limonoids, a dominant group of phytochemicals in the Rutaceae family, are known to exhibit several pharmacological activities. To identify natural products having efficacy against inflammatory bowel disease (IBD), we isolated 13 limonoids including a new compound, methyl sudachinoid A, from the seeds of *Citrus junos* and investigated their anti-inflammatory effects by assessing the expression of pro-inflammatory cytokines in lipopolysaccharide-stimulated RAW 264.7 mouse macrophages and HT-29 human colon epithelial cells. Our findings revealed that limonoids significantly downregulated the pro-inflammatory cytokines, such as interleukin (IL)-1β, IL-6, IL-8, tumor necrosis factor-α, and nuclear transcription factor κB. In particular, sudachinoid-type compounds, methyl sudachinoid A and sudachinoid B, and ichangensin-type compound, 1-*O*-methyichangensin downregulated the expression of pro-inflammatory cytokines more potently than other limonoids, nomilin and limonin, which have been previously reported to exhibit anti-inflammatory activities in other cells; nomilin and limonin were therefore employed as positive controls in this study. Herein, we reveal that the anti-inflammatory activities of limonoids including a new compound methyl sudachinoid A from *C. junos* were mediated via the downregulation of pro-inflammatory cytokines and these limonoids can be employed as potential therapeutic phytochemicals for IBD.

## 1. Introduction

Inflammation is a biological response to stimuli, such as pathogen infection, which serves as a major obstacle in the maintenance of a high quality of life [1]. Inflammatory reactions within the colon that might be caused by bacteria or viruses may initiate or accelerate the development or progression of colon cancer [2]. Inflammatory bowel disease (IBD) is characterized by chronic inflammation of the gastrointestinal tract. There are two principal types of IBDs: ulcerative colitis (UC) and Crohn’s disease (CD) [3]. To develop new treatments against these diseases, the mechanisms underlying the initiation, modulation, and progression of intestinal mucosal inflammation must be better understood. The chronic immune reaction in IBD may be regulated via increased secretion of pro-inflammatory cytokines caused by an improper response to the initial stimulating effect or impaired downregulation of cytokine secretion [4]. Inflammatory cytokines are rapidly induced and expressed in the early stages of a disease or injury in an antigen-independent manner [5]. The primary inflammatory cytokines are interleukin (IL)-1β, IL-6, and tumor necrosis factor (TNF)-α. IL-1β and TNF-α are pleiotropic cytokines that can alter the physiological and immunological responses and mediate the pathophysiological responses in different health conditions [6]. In addition, the activation of nuclear transcription factor κB (NF-κB) is noticeably stimulated in IBD patients and strongly affects the course of mucosal inflammation by inducing the expression of various pro-inflammatory genes [7].

The aberrant production of pro-inflammatory factors, such as chemokines (e.g., IL-8), often results in chronic inflammation [8]. The release of these cytokines results in the development of many inflammatory diseases, such as rheumatoid arthritis and IBD. As IL-8 functions as a significant regulatory factor within the tumor microenvironment [9], abrogating its activity represents a candidate therapeutic strategy for chronic inflammatory diseases. In the present study, the anti-inflammatory response of IL-8 was assessed in HT-29 human colon carcinoma cells.

Recently, several studies have sought to identify biologically active compounds from natural resources. Limonoids are plant-derived, highly-oxygenated, modified triterpenoids that have various pharmacological activities, such as antibacterial, antifungal, antimalarial, and anticancer effects [10]. Thus, there is increased interest in research on limonoids. The *Citrus* species from the Rutaceae family include coumarins, flavonoids, limonoids and carotenoids that have various pharmacologic activities [11]. Previously, we identified extracts with anti-inflammatory activity and fractions of coumarins using *Citrus junos* seeds. Additionally, we also attempted to discover novel anti-inflammatory phytochemicals from extracts that contain limonoids as their bitter constituents [12]. It was reported that some limonoids, such as obacunone, a limonoid abundantly distributed in citrus fruits, have anti-inflammatory activities [13]. In the present study, we sought to isolate limonoids and investigate their potential anti-inflammatory response by measuring the levels of inflammatory mediators, such as IL-1β and IL-6, and the activation of TNF-α, in RAW 264.7 mouse macrophage cells, and the level of IL-8 in HT-29 human colon carcinoma cells.

## 2. Results 

**Figure 1:**
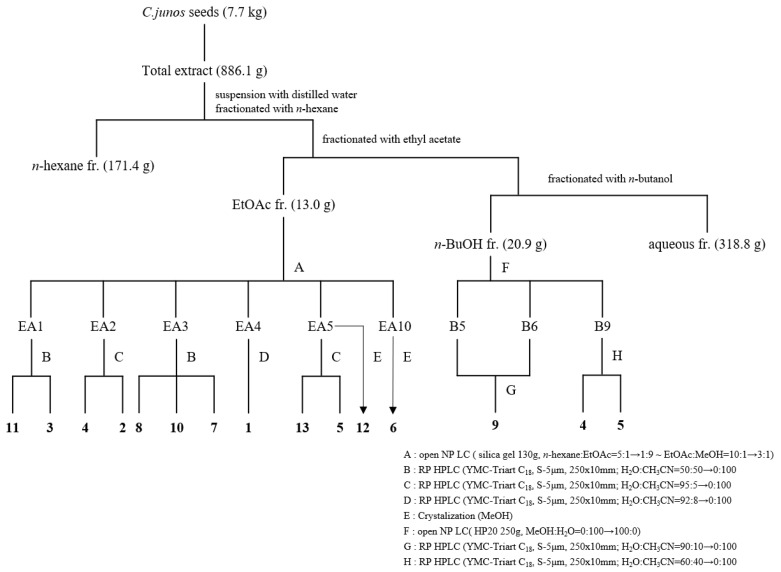
Isolation of limonoids from *C. junos* seeds. Twelve limonoids were isolated from the ethyl acetate fraction while three were isolated from the *n*-butanol fraction.

Compound **1** was isolated as a whitish amorphous powder from the EtOAc fraction of the *C. junos* seed extract (Figure 1). Based on positive HRESIMS, we determined that its molecular formula was C_27_H_36_O_9_ with *m*/*z* 505.2444 [M+H]^+^ (calculated for C_27_H_37_O_9_, 505.2422). Further, by normalizing the peak areas detected via ultra-performance liquid chromatography-photodiode array (UPLC-PDA) analysis, we calculated that its purity was 95%. Its ^1^H-NMR spectrum revealed the following results: one olefin proton signal [*δ*_H_ 7.61 (1H, d, *J* = 1.6 Hz, H-22)], three methines attached to the oxygen proton signal [*δ*_H_ 6.08 (1H, d, *J* = 1.6 Hz), 5.32 (1H, s, H-17), and 4.42 (1H, s, H-15)], two methoxy groups [*δ*_H_ 3.43 (3H, s, H-23) and 3.18 (3H, s, H-1)], two methine proton signals [*δ*_H_ 2.87 (1H, dd, *J* = 11.2, 6.4 Hz, H-9) and 2.54 (1H, m, H-5)], five methylene proton signals [*δ*_H_ 2.74 (1H, d, *J* = 14.8 Hz, H-6a), 2.20 (1H, dd, *J* = 14.6, 2.2 Hz, H-6b), 1.74 (1H, m, H-12), 1.71 (2H, m, H-11), and 1.23 (1H, m, H-12)], and six methyl groups [*δ*_H_ 1.23 (3H, s, H-2), 1.19 (3H, s, H-24), 1.16 (3H, s, H-25), 1.13 (3H, s, H-18), 1.11 (3H, s, H-19), and 1.03 (3H, s, H-26)].

**Figure 2:**
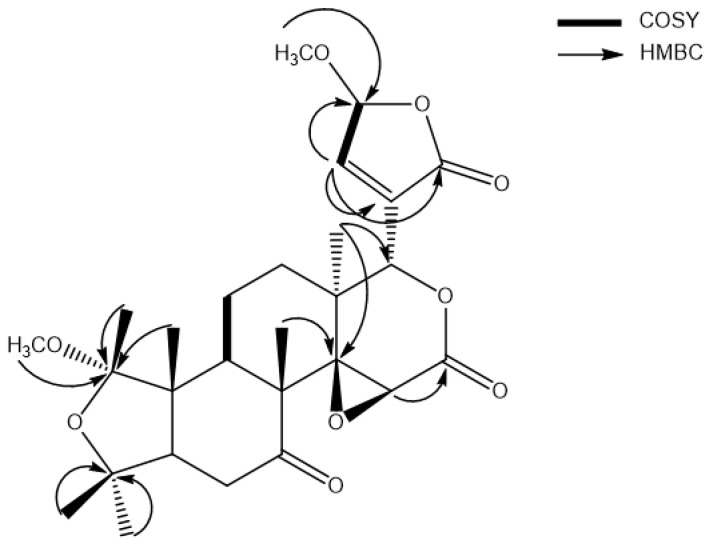
Key 2D NMR correlations for compound **1**.

The ^13^C-NMR spectrum of compound **1** contained resonances corresponding to three carbonyl carbon groups [*δ*c 208.4 (C-7), 169.0 (C-21) and 166.9 (C-16)], one double bond that might be the conjugated enone [*δ*_c_ 148.7 (C-22) and 135.5 (C-20)], two acetal carbon signals [*δ*_c_ 108.1 (C-1) and 103.5 (C-23)], three oxygenated methane carbon signals [*δ*_c_ 103.5 (C-23), 75.1 (C-17), and 55.6 (C-15)], two methoxy groups [*δ*c 56.0 (C-OCH_3_) and 48.2(C-OCH_3_)], and six methyl carbon signals [*δ*_c_ 31.6 (C-25), 23.6 (C-26), 18.9 (C-24), 18.4 (C-18), 17.6 (C-2), and 14.5 (C-19)]. In the H-C multiple bond correlation (HMBC) spectrum, the correlation of *δ*_H_ 1.13 (H-18) with *δ*_c_ 69.3 (C-14) and 75.1 (C-17); *δ*_H_ 3.43 (OCH_3_) with *δ*_c_ 103.5 (C-23); *δ*_H_ 7.61 (H-22) with *δ*_c_ 103.5 (C-23), 135.5 (C-20), and 169.0 (C-21); *δ*_H_ 4.42 (H-15) with *δ*_c_ 166.9 (C-16); *δ*_H_ 1.19 (H-24) with *δ*_c_ 69.3 (C-14); *δ*_H_ 1.11 (H-19), 1.23 (H-2), and 3.18 (OCH_3_) with *δ*_c_ 108.1 (C-1); and *δ*_H_ 1.16 (H-25) and 1.03 (H-26) with *δ*_c_ 79.4 (C-4) confirmed the structure of compound **1**. Further validation of the structure was achieved with the ^1^H-^1^H  correlation  spectroscopy  (COSY) correlations of *δ*_H_ 7.61 (H-22) with *δ*_H_ 6.08 (H-23) and *δ*_H_ 2.87 (H-9) with *δ*_H_ 1.71 (H-11) (Figure 2). Based on the above spectroscopic data, compound **1** was determined to be methyl sudachinoid A, a new compound isolated from a natural source.

**Figure 3:**
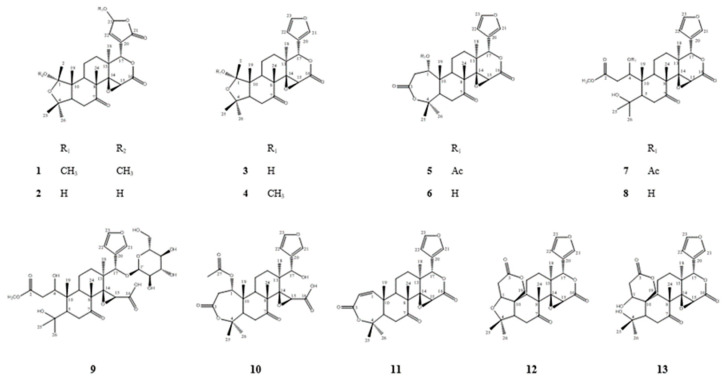
Structures of the limonoids isolated from *C. junos* seeds. Methyl sudachinoid A (**1**), sudachinoid B (**2**), ichangensin (**3**), 1-*O*-methylichangensin (**4**), nomilin (**5**), deacetylnomilin (**6**), methylnomilinate (**7**), methyldeacetylnomilinate (**8**), deacetylnomilinic acid-17-*O*-glucopyranoside (**9**), nomilinate A ring lactone (**10**), obacunone (**11**), limonin (**12**), and ichangin (**13**).

By comparing these spectroscopic data to those obtained in previously reported studies, the following 12 known compounds were identified: sudachinoid B (**2**), ichangensin (**3**), 1-*O*-methyichangensin (**4**), nomilin (**5**), deacetylnomilin (**6**), methylnomilinate (**7**), methyldeacetylnomilinate (**8**), deacetylnomilinic acid-17-*O*-glucopyranoside (**9**), nomilinate A ring lactone (**10**), obacunone (**11**), limonin (**12**), and ichangin (**13**) (Figure 3) [14,15,16,17,18,19,20,21].

**Figure 4:**
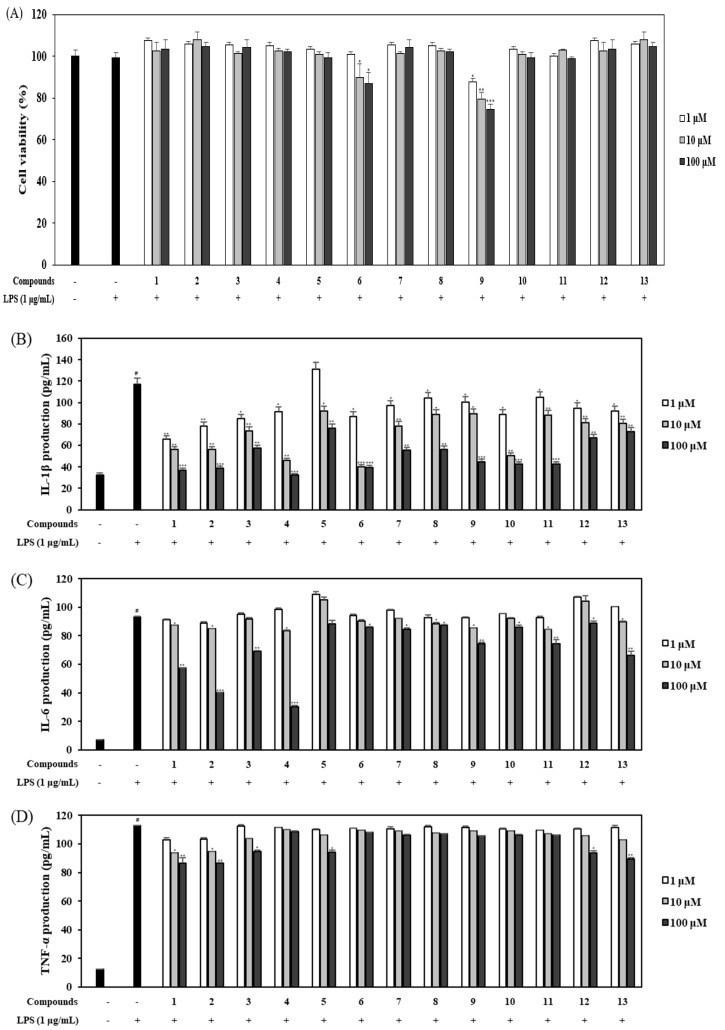
Effect of limonoids on the cell viability (**A**) and LPS-induced pro-inflammatory cytokine production (**B**–**D**). RAW 264.7 cells pretreated with compounds **1**–**13** (1, 10, and 100 µM) for 2 h were stimulated with 1 µg/mL LPS for 18 h. Cell viability was determined via MTT assay. The amount of IL-1β (**A**), IL-6 (**B**), and TNF-α (**C**) in the culture medium was measured with ELISA kits. The values are expressed as mean ± standard deviation of three individual experiments. * *p* < 0.05, ** *p* < 0.01, *** *p* < 0.001, compared to the control group.

RAW 264.7 mouse macrophages were pretreated with the 13 limonoids at different concentrations (1, 10, and 100 µM) for 1 h before stimulation with LPS (1 µg/mL) for 18 h. The control group was not treated with LPS or limonoids. The potential viability of the cells treated with the isolated compounds was measured via MTT assay. Subsequently, the inhibitory effect of the 13 compounds on the viability of cells was analyzed. With the exception of compound **9**, none of the limonoids isolated from *C. junos* seeds were cytotoxic (Figure 4A). Cellular exposure to LPS is known to result in the secretion of different inflammatory cytokines, such as IL-1β, IL-6, and TNF-α, leading to the amplification and initiation of inflammatory responses. To investigate the potential inhibitory effect of the inflammatory signaling in LPS-treated macrophages, we measured the production of IL-1β, IL-6, and TNF-α by ELISA. Based on our findings, all limonoids inhibited the LPS-activated IL-1β production in a concentration-dependent manner (Figure 4B). Among them, compounds **1**–**4**, **6**,**7**, and **11** dramatically suppressed the production of IL-1β to less than 50% at concentrations of 10 and 100 μM. Especially at a very low concentration of 1 μM, compound **1** and **2** had the most potent effect on inhibition of IL-1β production in the LPS-stimulated mouse macrophages. Additionally, these compounds showed better inhibitory activities against IL-6 and TNF-α production than the other limonoids (Figure 4C,D). As shown in Figure 4C, 100 µM of compound **4** decreased the production of IL-6 to 73.5%; however, the anti-inflammatory effect of this compound on LPS-induced TNF-α production was not observed. Compounds **1** and **2** potently reduced the pro-inflammatory cytokines in LPS-stimulated macrophages at all concentrations.

**Figure 5:**
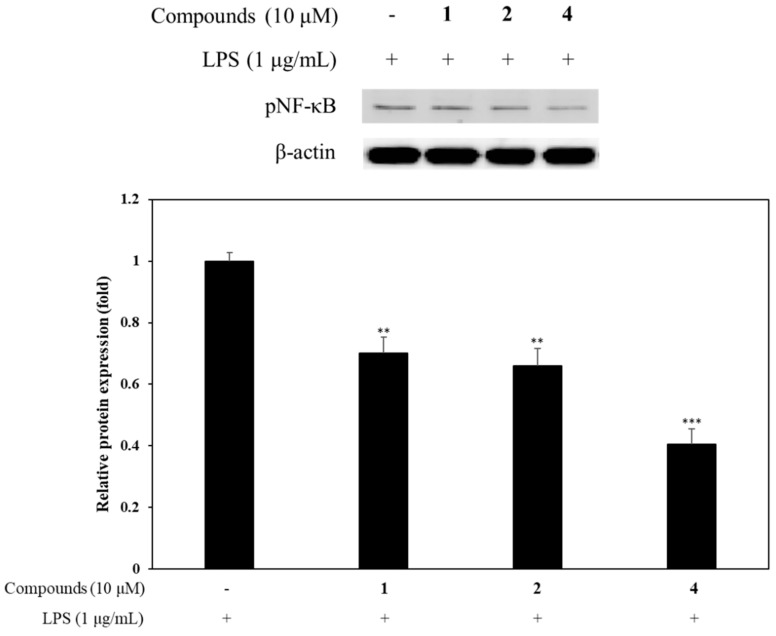
Effect of limonoids on NF-κB activation. RAW 264.7 cells were cultured in the presence of compounds **1**, **2**, and **4** (10 µM) for one hour and stimulated with LPS (1 µg/mL) for one hour. The levels of pNF-κB were detected by Western blot analysis. Relative density was calculated as the ratio of the expression levels of each protein with β-actin. The data are expressed as the mean ± SD (*n* = 3). ** *p* < 0.01 and *** *p* < 0.001, compared with LPS-stimulated group.

According to the results, compounds **1**, **2**, and **4** markedly affect the inflammatory cytokines. Western blot analysis was conducted at the treatment concentration of 10 µM for each compound to elucidate the anti-inflammatory effects via NF-κB signaling. All of these compounds downregulated the phosphorylation of NF-κB, stimulated by LPS (Figure 5). Compound **4** showed a greater inhibitory effect on LPS-induced pNF-κB activation than compounds **1** and **2**.

**Figure 6:**
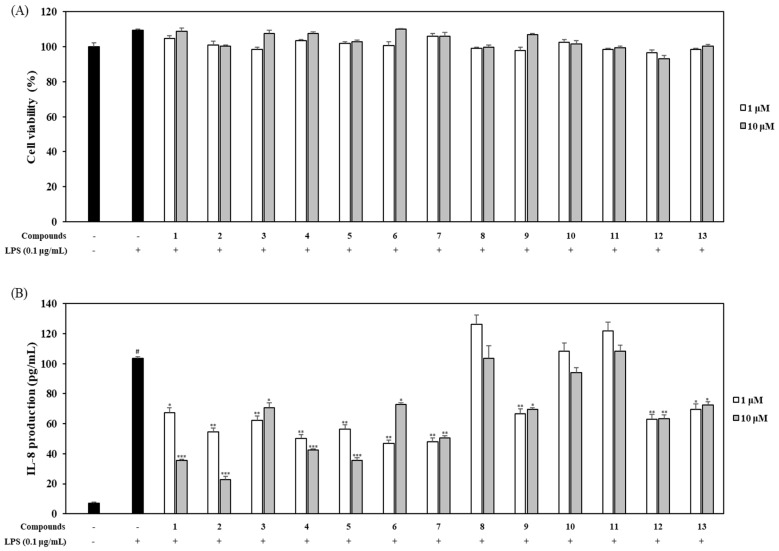
Effects of limonoids on the LPS-induced expression of IL-8 in HT-29 cells. HT-29 cells were treated with compounds **1**–**13** (1 and 10 µM) for 2 h and stimulated with 100 ng/mL LPS for 18 h. The viability of cells was then determined using an MTT assay (**A**) and the level of IL-8 in the culture media was measured with an ELISA kit (**B**). The values are expressed as mean ± standard deviation of three individual experiments. # *p* < 0.01, compared to the control group; * *p* < 0.05, ** *p* < 0.01, *** *p* < 0.001, compared to the LPS-treated group.

We assessed the effect of the 13 limonoids (1 and 10 µM) on the viability of LPS-stimulated HT-29 human colon epithelial cells. Our findings show that none of the limonoids exhibited any cytotoxicity. As a result, the 13 limonoids were employed in the subsequent experiments to investigate their anti-inflammatory activity (Figure 6A). IL-8 is a major chemoattractant and an activator of the neutrophils involved in mediating the immune response and promoting inflammation. The inhibitory effects of compounds **1**–**13** on IL-8 production were evaluated using ELISA kits. Compared to those in the untreated cells, the levels of IL-8 increased in colon epithelial cells subjected to LPS stimulation (100 ng/mL). Compounds **1**, **2**, **4**, and **5** inhibited IL-8 production in a concentration-dependent manner; the level of IL-8 in these cells was lower than that in LPS-stimulated cells (Figure 6B). In particular, 10 µM of compounds **1** and **2** resulted in potent anti-inflammatory activities (35.6 pg/mL and 22.8 pg/mL, respectively).

## 3. Discussion

Inflammation is a major global health problem that requires urgent management through the development of novel and efficacious therapeutic strategies. In recent years, there has been renewed interest in studying the mechanisms underlying inflammation as a basis for drug development [22]. Among the inflammatory diseases, IBDs, which are chronic disorders characterized by inflammation of the gastrointestinal tract, are being increasingly reported around the world [23]. An analysis of the inflamed mucosa from patients with UC and CD revealed that the pathogenesis of IBDs is related to the enhanced expression of pro-inflammatory cytokines such as IL-1 β, IL-6, TNF-α and IL-8, which induce other mediators that act on the inflammatory tissue, ultimately enhancing the inflammatory response [24,25]. Currently, anti-TNF therapies such as biosimilar anti-TNF monoclonal antibodies are widely used to treat IBD; however, these treatments are limited by factors such as adverse effects [26]. Therefore, we attempted to identify a novel agent capable of inhibiting the expression of pro-inflammatory cytokines, from a natural source.

Limonoids are highly oxygenated, secondary metabolites of the terpenoid class that are dominant in the Meliaceae and Rutaceae families [9]. Citrus fruits have an abundance of limonoids, and these compounds are mainly bitter in taste. Their prototypical structure either contains or is derived from a precursor possessing a 4,4,8-trimethyl-17-furanylsteroid skeleton. Citrus limonoids contain a furan ring and oxygen-containing functional group at C-3, C-4, C-7, C-16, and C-17, thereby displaying structural variations [27]. Limonoids also exhibit a wide range of biological properties such as antibacterial, antifungal, antimalarial, anti-viral, and anticancer activities [6]. Many studies have reported the chemical properties and pharmacological activities of limonoids, including their structure–activity relationship [27]. Recently, the anti-inflammatory activities of limonoids and limonoid glucosides were reported [28]. Therefore, we sought to determine the effects of the limonoids isolated from *C. junos* seeds on inflammatory responses using two in vitro models, RAW 264.7 mouse macrophage cells and HT-29 colon epithelial cells, which can simulate the characteristics of intestinal epithelial cells in IBD.

RAW 264.7 cells were treated with limonoids for 2 h before stimulation with 1 μg/mL LPS for 18 h. With the exception of compound **9**, none of the limonoids exerted any significant cytotoxicity at concentrations of 1, 10, and 100 μM (Figure 4A). However, the LPS-activated macrophages produced enhanced levels of pro-inflammatory cytokines such as IL-1β, IL-6 and TNF-α. The increased secretion of IL-1β, IL-6 and TNF-α has been demonstrated in experimental colitis models and in patients with IBD [29]. A reduction in IL-1β, IL-6 and TNF-α signaling was found to be effective at inhibiting macrophages in chronic intestinal inflammation, thereby indicating that IL-1β, IL-6 and TNF-α are potential therapeutic targets in IBD [30,31]. Pro-inflammatory cytokine levels are also enhanced in IBD intestinal cells [25]. To confirm the anti-inflammatory activity of limonoids, we assessed the LPS-induced production of IL-1β, IL-6 and TNF-α using ELISA kits. IL-1β plays a pivotal role in the regulation of immunity and inflammatory response. Additionally, it might play an important role in the pathogenesis of IBD by virtue of its pro-inflammatory and immunological activities [24]. Compounds **1** and **2**, which are sudachinoid-type limonoids, inhibited IL-1β production in a concentration-dependent manner (Figure 4B). This inhibition was found to be more potent than that induced by nomilin (**5**) and limonin (**12**), which were previously reported to have anti-inflammatory activities and were thus employed as the positive controls in this study [32,33]. Methyl sudachinoid (**1**; 1 μM), a new compound, dramatically suppressed IL-1β production to 60.3%. As compounds **3** and **4**, which are ichangensin-type limonoids, participated in the downregulation of IL-1β (71.2% and 83.8% at the high concentration of 100 µM), they might serve as therapeutic candidates for the treatment of IBD.

High mucosal secretion of IL-6 and TNF-α in IBD could be caused by infiltrating macrophages, which have been found to migrate in large numbers into the stimulated mucosal and intestinal lumen during UC and CD [4]. Similar to IL-1β production, compounds **1**-**4** reduced IL-6 production (47.7%, 63.9%, 36.7%, and 73.5%, respectively) at a concentration of 100 µM. Additionally, the ichangin (**13**), seco-limonin, significantly decreased IL-6 production in LPS-induced RAW 264.7 cells (Figure 4C). Compounds **1**–**3**, **5**, **12**, and **13** weakly attenuated TNF-α expression (Figure 4D). Such findings demonstrate that the citrus limonoids evaluated in this study markedly affect the downregulation of the pro-inflammatory cytokines in the order, IL-1β, IL-6 and TNF-α, and sudachinoid-type limonoids exert more potent anti-inflammatory activities than other limonoids containing nomilin and limonin.

NF-κB is an important regulator of gene transcription involved in anti-inflammatory signaling pathways in the gut [34]. In IBD patients, NF-κB signaling is often dysregulated resulting in inflammation and its activation is associated with the rapid, acute production of various proinflammatory mediators, such as IL-1β, and IL-6 [35]. Sudachinoid-type limonoids (compounds **1** and **2**) and ichangensin-type limonoid (compound **4**) most potently suppressed the production of IL-1β and IL-6, respectively (Figure 4). Therefore, among limonoids, we investigated whether inhibitory activities of proinflammatory cytokines by compounds **1**, **2**, and **4** are related to NF-κB signaling in LPS-stimulated macrophages. As shown in Figure 5, compounds **1**, **2**, and **4** attenuated the phosphorylation of NF-κB at a low concentration of 10 µM. The results showed that compounds **1**, **2**, and **4** have anti-inflammatory activity via inhibition of NF-κB-mediated pro-inflammatory cytokines and *Citrus* limonoids can effectively treat IBD.

Herein, we assessed the effects of the limonoids isolated from *C. junos* by treating HT-29 cells with 1 and 10 μM (i.e., the low concentrations) of the compounds for 2 h followed by incubation with 0.1 μg/mL LPS for 18 h before the MTT assay. Based on our results, none of the limonoids affected the viability of the cells (Figure 6A). When exposed to LPS, HT-29 cells secreted substantial amounts of IL-8. Standard intestinal epithelial cells can secrete the potent chemoattractant, IL-8, and could contribute to inflammation as opposed to level mucosa [36]. As the inhibitors of the pro-inflammatory chemokine, IL-8, may be used to treat immune-associated diseases, such as IBD, we measured the LPS-stimulated expression of IL-8 by treating HT-29 cells with limonoids. Ten limonoids (**1**–**7**, **9**, **12**, and **13**) significantly suppressed the LPS-stimulated expression of IL-8 at the low concentrations of 1 and 10 μM. However, only compounds **1**, **2**, **4**, and **5** inhibited IL-8 expression in a concentration-dependent manner; the remaining six compounds resulted in similar or increased expression with an increase in concentration (Figure 6B). The sudachinoid-type limonoids **1** and **2** effectively decreased the expression level of IL-8 in the LPS-stimulated human colon epithelial cells, a finding similar to that of the anti-inhibitory effect of pro-inflammatory cytokines in LPS-induced mouse macrophages. It was recently shown that obacunone (**11**) has an effect on bowel disease in mice via downregulating inflammatory signaling and restoring disrupted epithelial barriers [13]. Therefore, we considered that citrus limonoids, newly elucidated in our study, are more potent against IBD than previously reported limonoids and more in vivo studies need to be carried out to confirm the therapeutic effects. 

In conclusion, we attempted to discover novel phytochemicals for the treatment of IBD. Accordingly, we evaluated the anti-inflammatory effects of limonoids isolated from *C. junos* by measuring the production of pro-inflammatory cytokines in LPS-stimulated cell lines. During the investigation, a new limonoid, methyl sudachinoid (**1**), was isolated from the EtOAc fraction of *C. junos* seeds in addition to 12 known limonoids. Among the different limonoids, the sudachinoid-type compounds **1** and **2**, and ichangesin-type compound **4** potently suppressed the expression of pro-inflammatory cytokines, IL-1 β, IL-6, TNF-α and IL-8. Herein, we revealed the prospects of other limonoids, such as ichangin, for medicinal and/or nutraceutical use in the treatment and/or protection against IBD. According to our results, limonoids, including the new compound, isolated from *C. junos*, can be employed as potential therapeutic phytochemicals against IBD. In this study, we revealed the anti-inflammatory activity of citrus limonoids, which is underpinned by the downregulation of pro-inflammatory cytokines.

## 4. Materials and Methods

### 4.1. Plant Extract Preparation

*C. junos* seeds were collected from Goheung, Jeollanam-do, Korea, in November 2017. Subsequently, the essential oil was extracted from pulverised *C.junos* seeds (7.7 kg) by supercritical extraction. Following the removal of the essential oil, *C.junos* seeds were extracted with methanol to obtain the total extract (886 g), which was divided into *n*-hexane (171.4 g), EtOAc (13.0 g), *n*-BuOH (20.9 g), and distilled water (318.8 g) fractions [11].

### 4.2. Isolation of Limonoids from the Fractions

The EtOAc fraction was separated into 16 subfractions (EA1 to EA16) by silica gel column chromatography using a gradient solvent (*n*-hexane: EtOAc = 5:1 → 100% MeOH). Compounds **3** (*t_R_* 17.88, 23.2 mg) and **11** (*t_R_* 41.12, 1.8 mg) were obtained from EA1 by reverse phase (RP) high performance liquid chromatography (HPLC) (YMC-Triart, C_18_ column, 250 × 10 mm, CH_3_CN:H_2_O = 50:50 → 0:100). The EA2 fraction yielded compounds **2** (*t_R_* 32.92, 1.1 mg) and **4** (*t_R_* 48.04, 2.2 mg) when subjected to RP HPLC (YMC-Triart, C_18_ column, 250 × 10 mm, CH_3_CN:H_2_O = 5:95 → 100:0). The EA3 fraction yielded compounds **7** (*t_R_* 54.82, 7.6 mg), **8** (*t_R_* 46.96, 2.3 mg), and **10** (*t_R_* 51.56, 1.7 mg) when subjected to RP HPLC (YMC-Triart, C_18_ column, 250 × 10 mm, CH_3_CN:H_2_O = 50:50 → 100:0). The EA4 fraction yielded compound **1** (*t_R_* 49.15, 0.8 mg) when subjected to RP HPLC (YMC-Triart, C_18_ column, 250 × 10 mm, CH_3_CN:H_2_O = 8:92 → 100:0). The EA5 fraction yielded purified compound **12** through crystallization (MeOH), and compounds **5** (*t_R_* 33.84, 8.9 mg) and **13** (*t_R_* 29.94, 2.7 mg) when subjected to RP HPLC (YMC-Triart, C_18_ column, 250 × 10 mm, CH_3_CN:H_2_O = 5:95 → 100:0). The EA10 fraction yielded compound **6** through crystallization (MeOH). The *n*-BuOH fraction was separated into 12 subfractions (B1-B12) by Diaion HP20 column chromatography using 0%, 20%, 40%, 60%, 80%, and 100% MeOH. The B5 fraction was separated into 10 subfractions (B5-1 to B5-10) by silica gel column chromatography using a gradient solvent (chloroform:MeOH:H_2_O = 200:4:1 → 100% MeOH). Compound **9** (*t_R_* 24.64, 4.5 mg) was obtained from B5-7 by RP HPLC (YMC-Triart, C_18_ column, 250 × 10 mm, CH_3_CN:H_2_O = 10:90 → 100:0). Compounds **4** (*t_R_* 15.81, 2.4 mg) and **5** (*t_R_* 29.11, 2.5 mg) were obtained from the B9 fraction via RP HPLC (YMC-Triart, C_18_ column, 250 × 10 mm, CH_3_CN:H_2_O = 40:60 → 100:0). The basic schemes depicting the isolation process and structure are shown in Figure 1 and Figure 2, respectively.

Methyl sudachinoid A (**1**): whitish amorphous powder; [α]$\overset{25}{D}$: 23.7 (*c* 0.4 MeOH); ^1^H-NMR (400 MHz, DMSO-*d*_6_): *δ*_H_ 7.61(1H, d, *J* = 1.6 Hz, H-22), 6.08(1H, d, *J* = 1.6 Hz, H-23), 5.32(1H, s, H-17), 4.42(1H, s, H-15), 3.43(3H, s, OCH_3_), 3.18(3H, s, OCH_3_), 2.87(1H, dd, *J* = 11.2, 6.4 Hz, H-9), 2.74(1H, d, *J* = 14.8 Hz, H-6a), 2.54(1H, m, H-5), 2.20(1H, dd, *J* = 14.6, 2.2 Hz, H-6b), 1.74(1H, m, H-12), 1.71(2H, m, H-11), 1.23(3H, s, H-2), 1.23(1H, m, H-12), 1.19(3H, s, H-24), 1.16(3H, s, H-25), 1.13(3H, s, H-18), 1.11(3H, s, H-19), 1.03(3H, s, H-26); ^13^C-NMR (100 MHz, DMSO-*d*_6_): *δ*_C_ 208.4(s, C-7), 169.0(s, C-21), 166.9(s, C-16), 148.7(d, C-22), 135.5(s, C-20), 108.1(s, C-1), 103.5(d, C-23), 79.4(s, C-4), 75.1(d, C-17), 69.3(s, C-14), 56.0(q, OCH_3_), 55.6(d, C-15), 52.5(d, C-5), 49.5(s, C-8), 48.9(s, C-10), 48.2(q, OCH_3_), 40.5(s, C-13), 39.7(d, C-9), 36.6(t, C-6), 31.6(q, C-25), 26(t, C-12), 23.6(q, C-26), 18.9(q, C-24), 18.4(q, C-18), 17.6(q, C-2), 15.6(t, C-11), 14.5(q, C-19). High-resolution electrospray ionization mass spectrometry (HRESIMS) *m/z* 505.2444 [M+H]^+^ (calcd. C_27_H_37_O_9_, 505.2422). The spectra are available as Supplementary Materials (Figure S1–S7).

### 4.3. Cell Culture

HT-29 human colon epithelial cells and RAW 264.7 mouse macrophage cells were obtained from the Korean Cell Line Bank (Seoul, Korea) and cultured in Dulbecco’s modified Eagle’s medium (DMEM) (Hyclone, Logan, UT, USA) containing 10% heat-inactivated fetal bovine serum (FBS) (Hyclone, Logan, U.S.A.), 100 μg/mL streptomycin, and 100 IU/mL penicillin. The cells were incubated as monolayers for 72 h at 37 °C in a humidified environment containing 5% CO_2_.

### 4.4. Determination of Cytotoxicity by MTT (3-[4,5-Dimethyl-2-Thiazolyl]-2,5-Diphenyl Tetrazolium Bromide) Assay

The cells were seeded in 96-well plates (10^5^ cells/well) and maintained in DMEM containing 10% FBS for 24 h. Then, cells were treated with different concentrations of limonoids for 1 h prior to stimulation with lipopolysaccharide (1 µg/mL; LPS; Sigma-Aldrich, St. Louis, MO, USA) for 18 h. Cell viability was measured by an MTT assay, which involved the incubation of the cultured cells with MTT (0.05 mg/mL) at 37 °C for 4 h. After removal of the supernatants, the absorbance of the formazan solution was measured at 570 nm using a microplate reader.

### 4.5. Measurement of Pro-Inflammatory Cytokine Expression by Enzyme-Linked Immunosorbent Assay (ELISA)

RAW 264.7 and HT-29 cells were seeded in 96-well plates at a density of 1 × 10^5^ and 2 × 10^4^ cells/well, respectively, for 24 h. Thereafter, the cells were treated with the compounds for 2 h before stimulation with 1 μg/mL of LPS, followed by a 20 h incubation at 37 °C. We detected IL-1β(Invitrogen, Waltham, MA, USA), IL-6 and TNF-α (BD OptEIA^TM^, San Diego, CA, USA) levels in RAW 264.7 mouse macrophage cells, and the level of IL-8 (BD OptEIA^TM^, San Diego, CA, USA) in HT-29 cells using ELISA kits, as per the manufacturer’s protocol. Relative production (%) was calculated as ratio of the production levels of LPS treated and control group.

Relative production (%) = 100 × (sample treated group-control)/(LPS treated group-control)

### 4.6. Western Blot Analysis

Mouse macrophage were seeded at the density of 1 × 10^6^ cells/well in 6-well plates in culture medium for 24 h. Seeded cells were treated with compounds **1**, **2**, and **4** for 1 h, then stimulated with LPS (1 µg/mL). After 1 h, the cells were washed two times with cold phosphate-buffered saline (PBS) and whole cell lysates were extracted with protein extraction solution (proprep, iNtRON, Biotechnology, Daejeon, Korea). The protein concentration was determined by the Bradford reagent and Western blot analysis was done as described previously [37]. The primary (pNF-κB, β-actin) and secondary antibodies were diluted at 1:1000 and 1:2000, respectively.

### 4.7. Statistical Analysis

The data are presented as mean ± standard deviation (*n* = 3). Data analysis was carried out using one-way analysis of variance (ANOVA). *p*-values < 0.05 were considered to indicate significant differences.

## Supplementary Materials

The following are available online at https://www.mdpi.com/1422-0067/21/18/6963/s1, Figure S1: ^1^H-NMR spectra of compound 1; Figure S2: ^13^C-NMR spectra of compound 1; Figure S3: ^1^H-^1^H COSY spectrum of compound 1; Figure S4: HMQC spectrum of compound 1; Figure S5: HMBC spectrum of compound 1; Figure S6: DEPT spectrum of compound 1; Figure S7: HRESIMS of compound 1.
