# Supplementary material for: Sudachinoid- and Ichangensin-Type Limonoids from Citrus junos Downregulate Pro-Inflammatory Cytokines
Source: Int J Mol Sci. 2020 Sep 22;21(18):6963. doi: 10.3390/ijms21186963 (PMC7555237; doi:10.3390/ijms21186963)
Supplement: Supplementary file 1 [file ijms-21-06963-s001.pdf]

Supplementary data

Sudachinoid and Ichangensin Type Limonoids from *Citrus junos* down-Regulate Pro-inflammatory Cytokines

Jihun Shin, Hwa Young Song, Mina Lee

College of Pharmacy, Sunchon National University, 255 Jungangno, Suncheon-si 57922, Jeonnam, Republic of Korea

* Correspondence: minalee@sunchon.ac.kr; Tel. 82-61-750-3764 Fax. 82-61-750-3708

Supporting Information Available

Figure S1. 1H-NMR spectrum of compound 1
Figure S2. 13C-NMR spectrum of compound 1
Figure S3. 1H1H COSY spectrum of compound 1
Figure S4. HMQC spectrum of compound 1
Figure S5. HMBC spectrum of compound 1
Figure S6. DEPT spectrum of compound 1
Figure S7. HRESIMS of compound 1

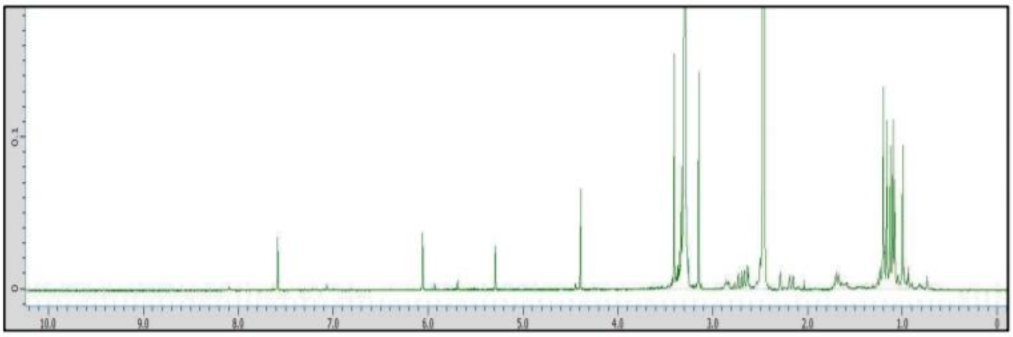

Figure S1. 1H-NMR spectra of compound **1**.

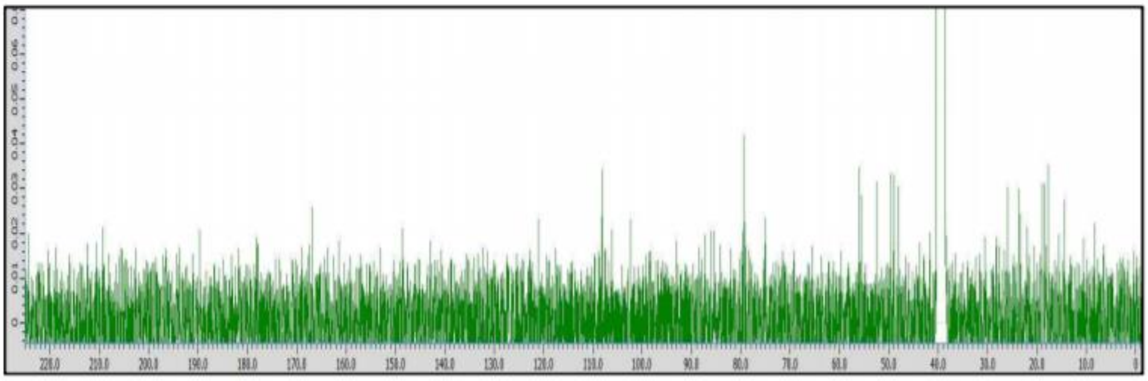

Figure S2. ^{13}C-NMR spectra of compound **1**.

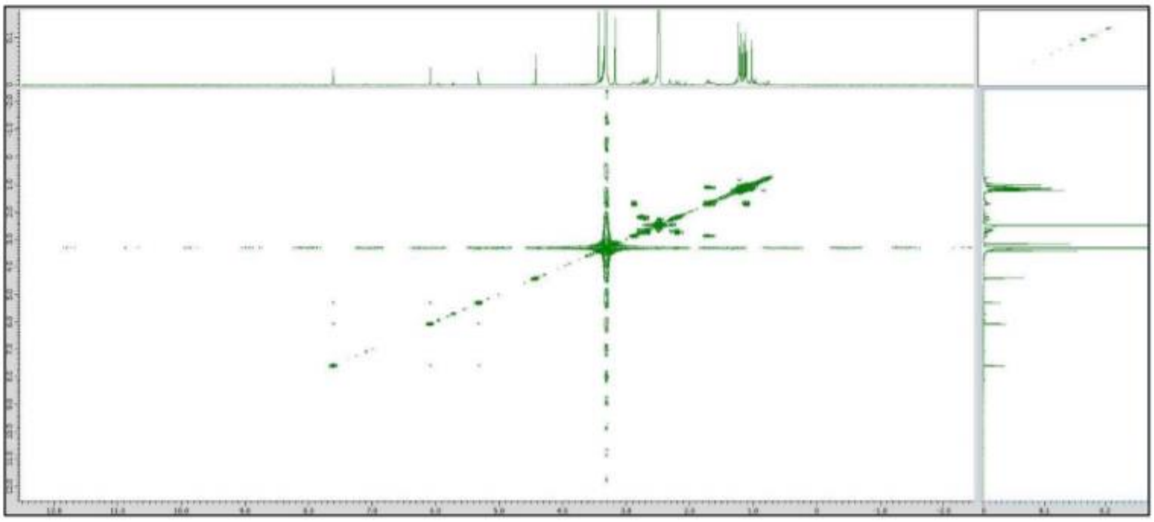

Figure S3. ^{1}H-^{1}H COSY spectrum of compound **1**.

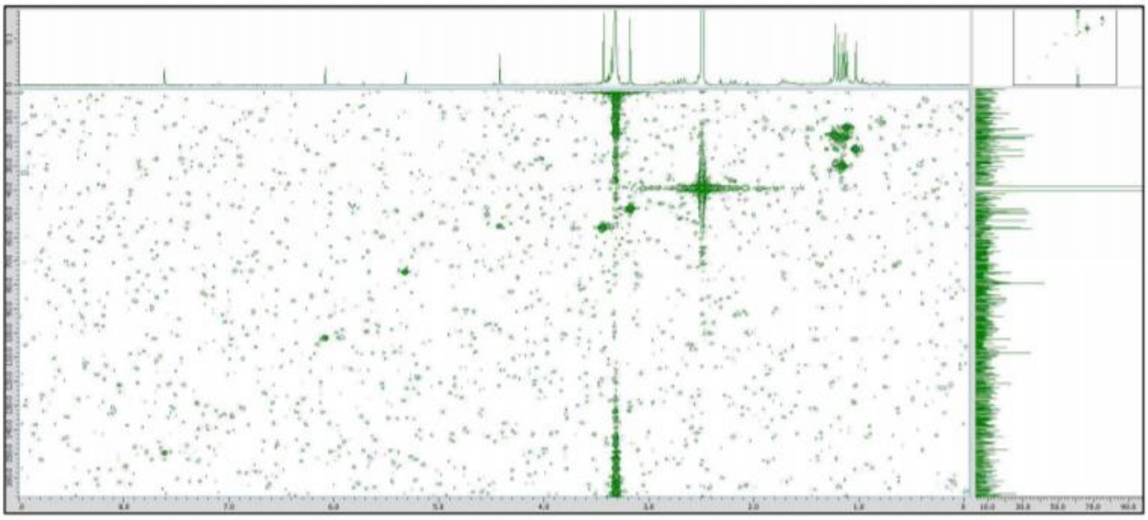

Figure S4. HMQC spectrum of compound **1**.

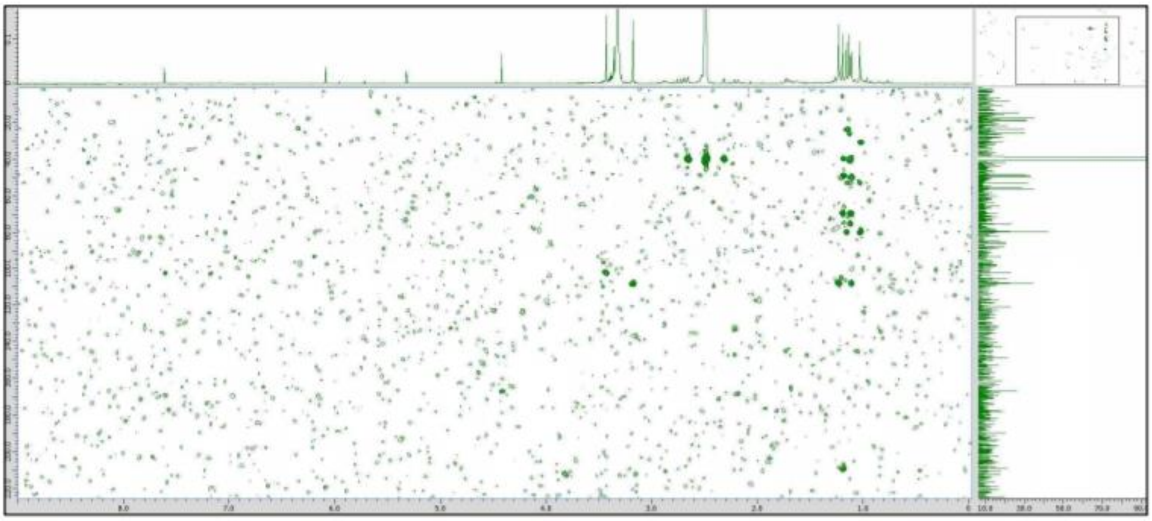

Figure S5. HMBC spectrum of compound **1**.

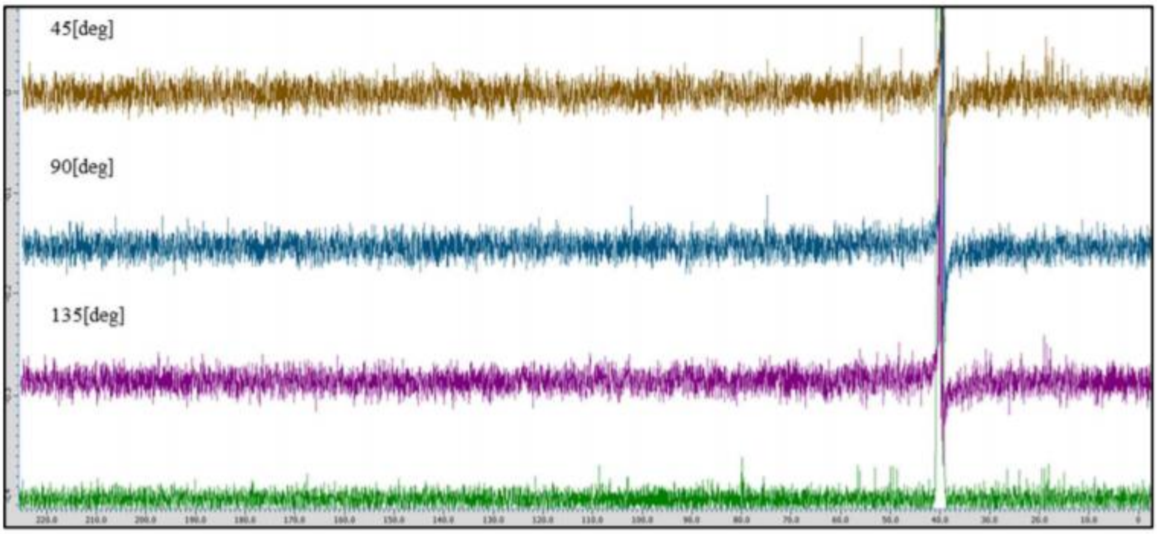

Figure S6. DEPT spectrum of compound **1**.

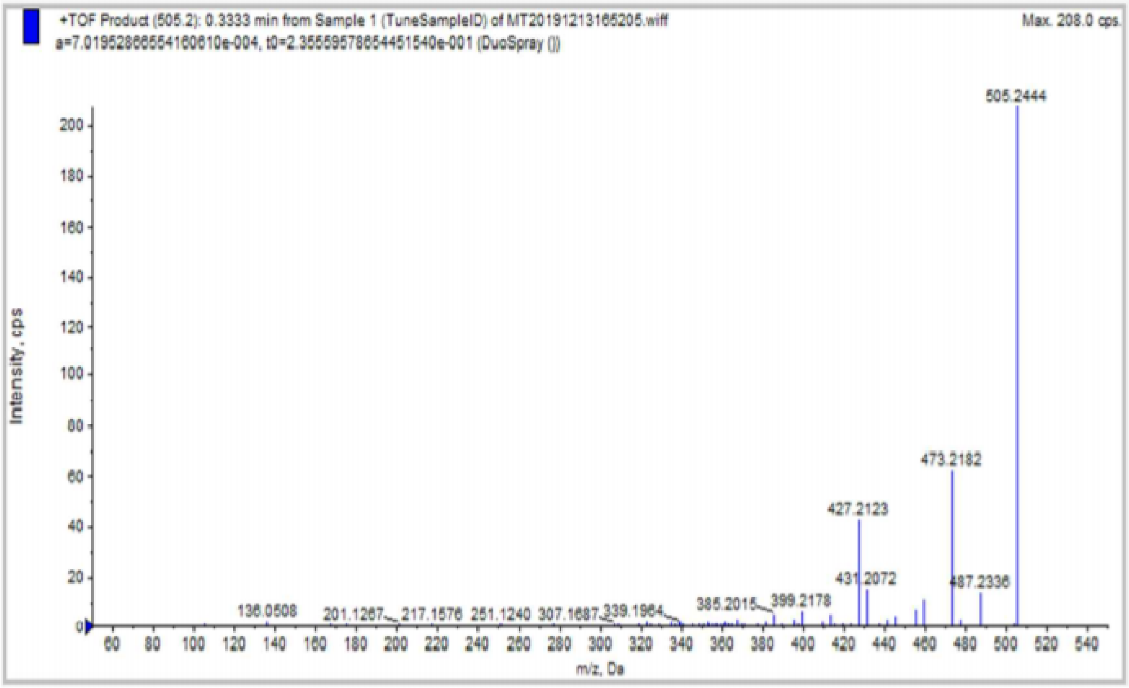

Figure S7. HRESIMS of compound **1**.
